# A comparison of neighbourhood level variation and risk factors for affective versus non-affective psychosis

**DOI:** 10.1016/j.schres.2022.05.015

**Published:** 2023-06

**Authors:** Peter Schofield, Henriette Thisted Horsdal, Jayati Das-Munshi, Malene Thygesen, Carsten Pedersen, Craig Morgan, Esben Agerbo

**Affiliations:** a School of Life Course and Population Sciences, Faculty of Life Sciences & Medicine, King's College London, London, United Kingdom; b Department of Psychological Medicine, Institute of Psychiatry, Psychology & Neuroscience, King's College London, London, United Kingdom; c Lundbeck Foundation Initiative for Integrative Psychiatric Research, iPSYCH, Aarhus, Denmark; d National Centre for Register-Based Research, Aarhus University, Aarhus, Denmark; e CIRRAU - Centre for Integrated Register-based Research at Aarhus University, Aarhus, Denmark; f South London & Maudsley NHS Trust; g ESRC Centre for Society and Mental Health, Kings College London; h National Institute for Health Research (NIHR) Mental Health Biomedical Research Centre at South London and Maudsley NHS Foundation Trust and King's College London

**Keywords:** Aetiology, Social determinants, Psychosis

## Abstract

**Background:**

Studies typically highlight area level variation in the incidence of non-affective but not affective psychoses. We compared neighbourhood-level variation for both types of disorder, and the specific effects of neighbourhood urbanicity and ethnic density, using Danish national registry data.

**Methods:**

Population based cohort (2,224,464 people) followed from 1980 to 2013 with neighbourhood exposure measured at age 15 and incidence modelled using multilevel Poisson regression.

**Results:**

Neighbourhood variation was similar for both disorders with an adjusted median risk ratio of 1.37 (95% CI 1.34–1.39) for non-affective psychosis and 1.43 (1.38–1.49) for affective psychosis. Associations with neighbourhood urbanicity differed: living in the most compared to the least urban quintile at age 15 was associated with a minimal increase in subsequent affective psychosis, IRR 1.13 (1.01–1.27) but a substantial increase in rates of non-affective psychosis, IRR 1.66 (1.57–1.75). Mixed results were found for neighbourhood ethnic density: for Middle Eastern migrants there was an increased average incidence of both affective, IRR 1.54 (1.19–1.99), and non-affective psychoses, 1.13 (1.04–1.23) associated with each decrease in ethnic density quintile, with a similar pattern for African migrants, but for European migrants ethnic density appeared to be associated with non-affective psychosis only.

**Conclusions:**

While overall variation and the effect of neighbourhood ethnic density were similar for both types of disorder, associations with urbanicity were largely confined to non-affective psychosis. This may reflect differences in aetiological pathways although the mechanism behind these differences remains unknown.

## 1 Introduction

Since the pioneering work of Faris and Dunham in Chicago in the 1930s there has been increasing interest in the role the neighbourhood environment plays in the aetiology of mental disorders, particularly psychosis. An often-overlooked aspect of this study is their central hypothesis that each mental disorder has a distinct social geography. They showed clear spatial patterns in rates of schizophrenia, associated both with levels of urbanicity and neighbourhood ethnic density. However, rates of affective (manic depressive) psychosis were reported to be randomly dispersed.

Subsequent studies appear to follow a similar pattern: showing heterogeneity in rates of *non-affective* psychosis between areas, beyond that which could be attributed to chance, but failing to show any corresponding heterogeneity in rates of *affective* psychosis (Giggs, 1986; Scully et al., 2004; Kirkbride et al., 2007a, Kirkbride et al., 2014). Similarly studies in Northern Europe, using large epidemiological datasets, have consistently shown clear urban/rural differences in rates of non-affective psychoses, such as schizophrenia, but have failed to show any comparable difference in rates of affective psychosis or bipolar disorder (Marcelis et al., 1998; Eaton et al., 2000; Mortensen et al., 2003; Pedersen and Mortensen, 2006; Laursen et al., 2007; Kroon et al., 2013; Vassos et al., 2015). Some therefore argue that these differences are evidence for the long disputed Kraepelinian dichotomy between the two types of disorder (Marcelis et al., 1998; Laursen et al., 2007; Vassos et al., 2015) and appear to promote differential etiological pathways (Kirkbride et al., 2007a).

However, despite these apparently consistent patterns, methodological shortcomings make it difficult to draw firm conclusions. Firstly, neighbourhood exposure is typically measured at diagnosis only, i.e. *after* illness onset (Scully et al., 2004; Kirkbride et al., 2007b, Kirkbride et al., 2014; Jongsma et al., 2018; Richardson et al., 2018), which makes it difficult to rule out reverse causality. For example, a change of address during illness or the prodromal phase might explain these apparent differences. Reverse causality may be less applicable to affective psychoses where, we argue, a typically older age of onset and a more common relapsing and remitting course could potentially promote greater housing stability and thus mitigate against ‘drift’ into urban areas (Morgan et al., 2014; Velthorst et al., 2017). It is therefore possible that the apparent specificity in spatial patterning shown in some cross-sectional studies could simply be an artefact of differences in course and onset. To our knowledge only two previous longitudinal studies have compared area level risk factors in this way – both concentrate on urbanicity alone and both show degree of urbanisation at birth to be more strongly related to non-affective compared to affective psychosis (Laursen et al., 2007; Vassos et al., 2015).

Secondly, it is possible that the relative rarity of affective psychosis means that a lack of evidence for neighbourhood differences in this disorder simply reflects a lack of statistical power. This may partly explain the stark contrast between the considerable research attention given to contextual factors relevant to schizophrenia in comparison with the relative scarcity of comparable research looking at bipolar disorder (Muntaner et al., 2004).

A further limitation is that most studies pay little attention to specific neighbourhood factors, other than urbanicity, which may differentiate between the two types of psychoses. For example, neighbourhood ethnic density has also been consistently shown to be associated with incidence of non-affective psychosis (Veling et al., 2006; Kirkbride et al., 2007a; Schofield et al., 2017b). While a wealth of studies in recent years have demonstrated clear associations with ethnic density and non-affective psychosis and also common mental disorders, affective psychosis has been largely neglected (Shaw et al., 2012; Bécares et al., 2018). This is an important omission as it has been proposed that ethnic density could help explain apparent area differences in rates of bipolar affective disorder among some ethnic groups (Lloyd et al., 2005).

While associations between some environmental factors and psychosis are well established, at least in large Northern European cohort studies, we still know little about why these patterns occur (March et al., 2008; van Os et al., 2010; Vassos et al., 2012; DeVylder et al., 2018). Comparing effects across different psychosis phenotypes, it is argued (van Os et al., 2016), may help to shed some light on possible mechanisms.

### 1.1 Aims of the study

To this end we set up a prospective study to compare the role of neighbourhood at age 15 on the subsequent incidence of affective and non-affective psychosis using Danish register data over a thirty-year period. We set out to assess differences in overall neighbourhood level variation between disorders as well as differences in associations with neighbourhood urbanicity and neighbourhood ethnic density.

## 2 Method

### 2.1 Sample

We looked at a whole population cohort, comprising all those born after 1965 and living in Denmark on their 15th birthday between 1st January 1980 and 31 December 2012. The cohort were followed from their 15th birthday until they either died, migrated, were diagnosed with a non-affective or affective psychosis or 1st of July 2013 (whichever came first).

The study used data collected under the Danish Civil Registration System whereby all Danish citizens are granted a unique personal identification number allowing data to be linked across a range of population registers, including: health, employment and residential data across the life course (Pedersen, 2011).

### 2.2 Measures

#### 2.2.1 Outcomes

Cohort data was linked to the Danish Psychiatric Central Register which covers all psychiatric in-patient admissions and, since 1995, all out-patient visits (Mors et al., 2011). Individuals were classified based on their discharge diagnosis. For non-affective psychoses (schizophrenia or a schizophrenia related disorder) we used the following ICD10 codes: F20-F29, and their equivalent ICD-8 codes: 295.x9, 296.89, 297.x9, 298.29–298.99, 299.04, 299.05, 299.09, 301.83. For affective psychoses (bipolar disorder with psychosis or depression with psychosis) we used the following ICD10 codes: codes F30.2, F31.2, F31.5, F31.6, F31.8, F32.3, F33.3 and their ICD-8 equivalents: 296, 298.0, 298.1. For non-affective psychosis the equivalent ICD-8 codes match those used in previous Danish register studies (Pedersen et al., 2014; Mok et al., 2016). For affective psychosis, relevant codes were derived in consultation with Professor Aksel Bertelsen (see Appendix 1) who, as international adviser to the WHO's Division of Mental Health, was responsible for preparing the Danish version of ICD-10 and played a key role in educating Danish psychiatrists in its use (Bertelsen, 2000; Munk-Jørgensen et al., 2020).

Date of diagnosis was defined as the date of first contact with the relevant diagnosis. In the main analysis, cases were censored where there had been a preceding diagnosis of another psychotic disorder so that we consider only the first of the two disorders. For example, when ascertaining cases of non-affective psychosis, if the first diagnosis was an affective psychosis then any subsequent diagnosis of non-affective psychosis was ignored. We also carried out a sensitivity analysis excluding cases where the diagnosis changed over a defined period, as outlined below.

#### 2.2.2 Neighbourhood exposures

Neighbourhood units were based on amalgamated Danish parishes derived for our previous study (Schofield et al., 2017, Schofield et al., 2017b) giving a total of 1167 parish units with a median population of 3600 residents. For the purposes of this analysis we defined neighbourhood as the neighbourhood in which the cohort member was resident on their 15th birthday. A measure of urbanicity was derived at parish level using population density (residents per km^2^) in the year the cohort member was 15. Neighbourhood ethnic density was calculated at parish level for African, European (non-Scandinavian) and Middle Eastern migrants using the proportion of people from the same migrant group, as the index cohort member, living in the area when the cohort member was 15. These groups were chosen as they are the largest migrant groups in Denmark (see Appendix 4 for a detailed list of countries in each category). In a previous study we found associations between neighbourhood urbanicity, ethnic density and psychotic illness were inter-related (Schofield et al., 2017b). To avoid confounding we therefore restricted our urbanicity analysis to native Danes only and for the ethnic density analysis we further adjusted for the effect of urbanicity.

#### 2.2.3 Other covariates

As well as adjusting for age, gender and period effects, we also adjusted for parental income at age 15 and parental psychiatric history. Parental income was defined as the combined parental gross annual income at age 15, divided into quartiles within each year (Petersson et al., 2011). Parental psychiatric history was defined as any psychiatric disorder in either parent prior to the cohort member's 15th birthday (Mors et al., 2011).

### 2.3 Statistical analysis

We used multilevel Poisson regression to model effects at three levels: 1) individuals nested within 2) calendar year (when aged 15) nested within 3) neighbourhoods (parish).

Analyses were adjusted for age, gender (plus age/gender interactions) and calendar time. Age and calendar time were included as time varying covariates, using the Lexis expansion method. Age was categorised as: 15–20, 20–24, 25–29, 30–34, 35–39, 40–44, 45–49, 50–54, and 55 or older. Calendar time was categorised into 5-year age bands, and 2-year age bands in the 1990s, to account for changes in Denmark from ICD-8 to ICD10 diagnostic systems. Person time was calculated as the number of days from the date of the cohort member's 15th birthday until they either died, migrated, were diagnosed with a non-affective or affective psychosis or 1st of July 2013 (whichever came first).

We assessed the extent to which each type of psychosis varied at neighbourhood level using the median rate ratio (MRR) which provides a single intuitive measure of neighbourhood variation (Austin et al., 2018). This is defined as the ratio between high and low risk areas when randomly picking out two areas, and can be conceptualised as the median increase when moving from one area to another area with a higher risk (Merlo et al., 2006). We modelled overall neighbourhood level variation in two stages: first, adjusting for the individual level factors age, gender and calendar time; and, second, further adjusting for ethnic group (using the above categories), parental income and parental psychiatric history.

Neighbourhood urbanicity at age 15 was categorised into quintiles, determined across the whole population, and we report here comparisons with the least urban quintile. Neighbourhood ethnic density was modelled as a cross-level interaction with the relevant migrant group. For example, for migrants from Africa we compare those living in an area with a greater or lesser proportion of other migrants from Africa. We are restricted here to reporting the overall trend across quintiles because for the African and Middle Eastern groups individual quintiles contained fewer cases than the permissible number set by statistics Denmark to safeguard anonymity. Ethnic density quintiles were determined across each specific migrant cohort so there would be at least some cases for the relevant migrant group within each quintile.

**Fig. 1:**
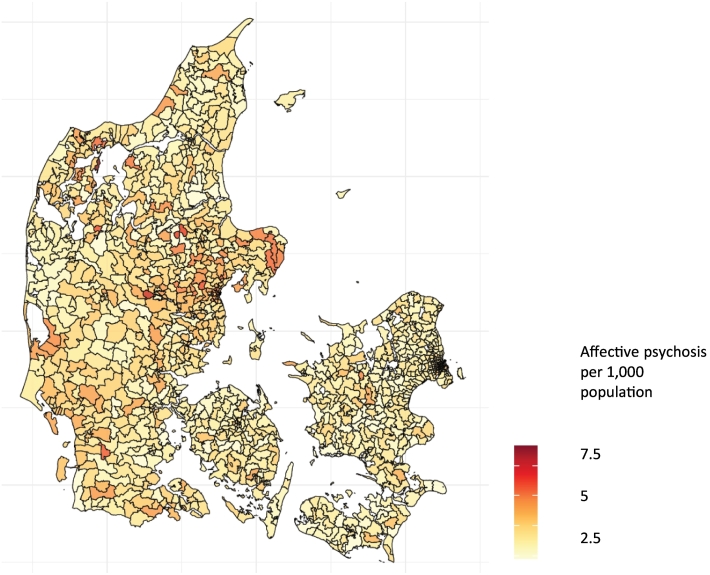
Cumulative prevalence of affective psychosis by parish in the study cohort.

**Fig. 2:**
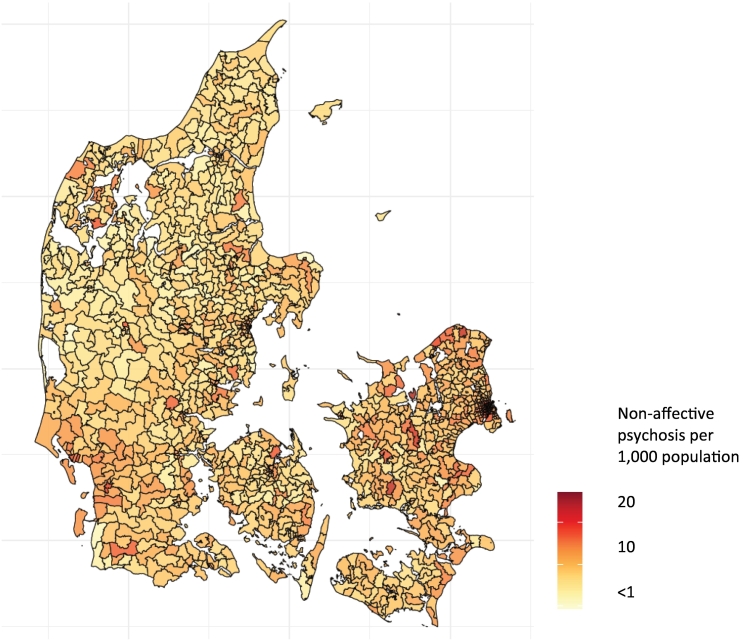
Cumulative prevalence of non-affective psychosis by parish in the study cohort.

We tested for over-dispersion by examining the distribution of both disorders and found no evidence for over-dispersion. For illustrative purposes we also provide maps comparing prevalence of both types of disorder in the final year of the cohort (Fig. 1, Fig. 2).

All analyses were conducted using Stata (version 15).

### 2.4 Sensitivity analysis

We also carried out a sensitivity analysis to test whether our results were sensitive to a change in diagnosis (Appendix 2 - Tables 5, 6 and 7). We took a five-year window after the first diagnosis of a psychotic illness and excluded any cases who subsequently received an alternative diagnosis of a different psychotic disorder during this period i.e. cases of non-affective psychosis were excluded if there was a subsequent diagnosis of affective psychosis, and vice-versa. We then re-ran the analysis and compared our results for this revised outcome measure.

We also carried out a further sensitivity analysis to test whether our results were sensitive to the diagnostic scheme used, specifically the change from ICD-8 to ICD-10. We therefore repeat our analysis this time looking at those diagnosed according to ICD-10 only – to see if our results are sensitive to the diagnostic scheme applied. We use a new entry point – beginning 1st January 1995 exactly one year after ICD-10 was first introduced in Denmark (Povl Munk-Jørgensen, 2009).

### 2.5 Ethical approval

Ethical approval is not required to use the register-based data in Denmark. However, access to use the data required the completion of a detailed application form from the Danish Data Protection Agency, the Danish National Board of Health and Statistics Denmark.

## 3 Results

### 3.1 Overall neighbourhood level variation

We first looked at overall variation in rates of subsequent psychotic disorder across neighbourhoods (where cohort members were living at age 15) (Table 1). We found neighbourhood level variation for both non-affective and affective psychoses. For example, the median risk ratio (MRR) for rates of non-affective psychoses across neighbourhoods, after adjusting for age, gender and calendar period, came to 1.37 (95% CI 1.34–1.39) and for affective psychosis this was slightly higher: MRR 1.43 (1.38–1.49). Further adjusting for ethnic group parental income and psychiatric history made little difference to these results although the variation in non-affective psychosis was slightly reduced MRR 1.30 (1.28–1.33).

**Table 1:** Variation in incidence of psychotic disorder across neighbourhoods.^a^

	Median risk ratio (95% CI)	
	Non-affective psychosis	Affective psychosis
Model 1^b^	1.37 (1.34–1.39)	1.43 (1.38–1.49)
Model 2^c^	1.30 (1.28–1.33)	1.43 (1.37–1.49)

a Where cohort member was resident at age 15.

b Adjusted for age, gender and calendar period.

c Adjusted for age, gender, calendar period, ethnicity, parental psychiatric history and parental income.

### 3.2 Neighbourhood urbanicity

We found that associations with neighbourhood urbanicity were largely confined to incidence of non-affective psychosis (Table 2). For example, we found evidence for a dose-response effect and a large increase in rates of non-affective psychosis in the most urban quintile, incidence rate ratio (IRR) 1.66 (1.57–1.75) compared to the least urban quintile. In contrast, for affective psychosis there was no evidence for a dose-response effect or positive association with any increase in urbanicity other than a small difference for those in the most urban quintile, IRR 1.13 (1.01–1.27).

**Table 2:** Incidence rate ratios of psychotic disorder by neighbourhood^a^ urbanicity (native Danes only).

Urbanicity (quintile)	Incidence rate ratio (95% CI)^b^	
	Non-affective psychosis	Affective psychosis
1 (lowest)	1	1
2	1.07 (1.01–1.13)	0.98 (0.88–1.09)
3	1.22 (1.15–1.29)	0.99 (0.88–1.11)
4	1.30 (1.23–1.38)	0.95 (0.85–1.08)
5 (highest)	1.66 (1.57–1.75)	1.13 (1.01–1.27)

a Where cohort member was resident at age 15.

b Adjusted for age, gender, calendar period, parental psychiatric history and income.

### 3.3 Neighbourhood ethnic density

We found a similar ethnic density trend associated with rates of both non-affective and affective psychosis for the African and Middle Eastern migrant groups (Table 3). For migrants from Africa, living in a lower African quintile neighbourhood at age 15 was associated with an increased incidence of both non-affective, IRR 1.22 (1.09–1.36), and affective psychosis, IRR 1.40 (0.98–1.96). For Middle Eastern migrants, a similar trend was observed. A decrease in ethnic density quintile was associated with an increase in incidence of both non-affective, IRR 1.14 (1.05–1.24), and affective psychosis, IRR 1.54 (1.20–1.99). Conversely, for migrants from elsewhere in Europe (non-Scandinavian), a decrease in ethnic density quintile for this group was associated with an increase in incidence of non-affective, IRR 1.14 (1.08–1.21), but not affective psychosis IRR 1.03 (0.88–1.20).

**Table 3:** Incidence rate ratios of psychotic disorder by neighbourhood^a^ ethnic density for each migrant group.

Ethnic density trend (across quintiles) for each country of origin^c^	Incidence rate ratio (95% CI)^b^	
	Non-affective psychosis	Affective psychosis
Africa	1.22 (1.09–1.36)	1.38 (0.98–1.96)
Europe	1.14 (1.08–1.21)	1.04 (0.88–1.20)
Middle East	1.14 (1.05–1.24)	1.54 (1.20–1.99)

a Where cohort member was resident at age 15.

b Adjusted for age, gender, calendar period, parental psychiatric history and income and neighbourhood urbanicity.

c Measured as trend over decreasing ethnic density quintiles i.e. the average change in psychosis incidence corresponding to a change from one ethnic density quintile to the next lower density quintile.

### 3.4 Sensitivity analyses

We also re-ran the analysis excluding any cases where the diagnosis changed within a five-year period from the initial diagnosed psychotic disorder. Restricting cases to only those with a more stable diagnosis, in this way, made no appreciable difference to the above study results (see supplementary tables in online Appendix 2). We also re-ran the analysis looking at those diagnosed according to ICD-10 only to see if our results were sensitive to the diagnostic scheme applied. As our results show (Appendix 3) this made no tangible difference to our study findings.

## 4 Discussion

### 4.1 Summary of the results

We found substantial neighbourhood variation in rates of both affective and non-affective psychosis implying heterogeneity in rates of both types of disorder at neighbourhood level. However, when we looked at specific spatial factors the results were mixed. Neighbourhood urbanicity was primarily associated with non-affective psychosis only while neighbourhood ethnic density was typically associated with both.

### 4.2 Strengths and limitations

This is the most comprehensive study to date to investigate area level differences between different types of psychotic disorder. We used whole population data, including all inpatient and outpatient psychiatric records collected across the lifespan, which enabled us to determine residential neighbourhood well in advance of illness onset. We present evidence that urbanicity is only very weakly associated with rates of affective psychosis despite this part of the study being well powered to detect the presence of an effect. For neighbourhood ethnic density, we show the presence of an effect for both types of disorder despite this part of the analysis having lower statistical power to detect an association.

A limitation of the study was the use of ICD codes to distinguish psychotic illness. While precise distinctions in psychiatric nosology are problematic at best, our task was further complicated by the shift from ICD-8 to ICD-10 during the time period of our cohort. Our definition of affective psychosis, as distinct from bipolar disorder and major depression disorder, could only be approximate given the limitations of the ICD system (see Appendix 1). For example, it is not possible to exactly specify affective psychosis with or without psychotic symptoms (both in ICD-8 and ICD-10). For ICD-8 diagnoses, in particular, patients from the sample diagnosed before 1995 may be classified under the broad category of affective psychosis without having psychotic symptoms. However, we were able to show in our sensitivity analysis, that excluding ICD-8 cases made no difference to our overall results. For ICD-10, two codes (F31.6 mixed bipolar affective disorder and F31.8 other bipolar affective disorder) may or may not reflect a mood disorder with psychotic features. While we were unable to distinguish particular ICD-10 codes in our analysis, we were able to look separately at their use in the Danish psychiatric register over the time period of our study. We found that only 5.4% were defined according to these two codes (3.2% using code F31.6 and 1.8% using code F31.8). Given that these codes are so rarely used this is unlikely to have had any bearing on our study results.

We do, though, acknowledge that it would be helpful for future studies to look at sub-groups within these broader diagnostic categories. For example it would be useful to be able to differentiate more clearly between bipolar disorder with and without psychosis, as others have suggested (Kaymaz et al., 2006). However, an analysis at this level of detail raises significant challenges in terms of both statistical power and the limitations of the ICD system as it applies to this particular cohort.

Furthermore, while we adjust for parental psychiatric disorder we cannot rule out the possibility of residual confounding where undiagnosed or sub-threshold mental disorder experienced by the parent influences the type of neighbourhood where the subject is living at age 15. We have also further adjusted for parental socio-economic background based on parental income. However, we cannot rule out the possibility that other socio-environmental factors may also be important. It is also important to note that the study is reliant on health records alone and may not include less severe cases. However, we have no reason to believe that diagnosis is not consistent across Denmark and there is no evidence for area differences in the threshold for admission with a psychotic disorder (Pedersen and Mortensen, 2001).

### 4.3 Comparison with previous studies

Our findings, showing overall area-level variation for both disorders, contrast with previous large-scale cross-sectional studies which report area-level variation as specific to non-affective psychosis alone. For example, one study analysed psychosis incidence data collected for the Aetiology & Ethnicity in Schizophrenia and Other Psychoses [ÆSOP] study covering South East London (Kirkbride et al., 2007a). This found significant heterogeneity, across census wards, in rates of non-affective psychosis, after adjusting for age, sex and ethnicity, but found no difference in rates of affective psychosis. The study authors also conducted a later study using health referrals data collected for East London and again, once individual level factors were accounted for, they found that spatial variation was specific to non-affective psychosis only (Kirkbride et al., 2014). These studies were, however, relatively small scale, limited to cross-sectional data only and based on much broader diagnostic categories including, for example, major depressive disorder under the affective psychosis category. In particular it is possible that differences from our study results may be due to a lack of power and, or, an inability to distinguish the differential effects of social drift.

Our findings for the association with urbanicity, on the other hand, show a similar pattern to that demonstrated by other large scale cohort studies, including previous studies using similar Danish cohort data (Laursen et al., 2007; Vassos et al., 2015).

Very few studies in recent years have looked at neighbourhood ethnic density and rates of affective psychosis. One exception was a cross-sectional analysis of health referral data across a large rural area in England and, interestingly, the authors report that higher ethnic density was associated with lower rates of *affective* psychosis but made no difference to rates of non-affective psychosis (Richardson et al., 2018). However, again it is possible this was an artefact of social drift as neighbourhood exposure was measured at the time of first presentation. It is also possible that this reflects their much broader categorisation of affective psychosis. Another study looked at Iraqis living in Iraqi immigrant enclaves in Sweden and found this made no difference to rates of non-affective and affective psychosis (Mezuk et al., 2015). However, Swedes living in these areas were more likely to have higher rates of both disorders, although the latter is likely to be related to social drift as this study was also based on cross-sectional data only. Our findings using a longitudinal cohort showing that ethnic density is relevant to both affective and non-affective psychosis would appear to refute the argument that affective psychosis may be less influenced by the wider social environment for minority ethnic populations, as some have suggested (Tortelli et al., 2015).

### 4.4 Interpretation

Our results show that neighbourhood at age 15 is relevant to later incidence of both types of psychotic disorder, even after adjusting for a wide range of possible parental confounders. Which neighbourhood characteristics are important is a more complex question. The differential associations with neighbourhood urbanicity and ethnic density we found, may reflect different underlying mechanisms. For example, one possible explanation for the differential effect of neighbourhood urbanicity is the mediating role of developmental deficits. Developmental deficits are commonly associated with non-affective psychosis but much less so with affective psychosis (Rietschel et al., 2009; Arango et al., 2014; Bora, 2016). It has been argued that, while both psychosis phenotypes share an underlying genetic propensity, environmental risk factors such as urbanicity may impair cognitive development and thus predispose to non-affective psychosis (Walker et al., 2002; Murray et al., 2004; Parellada et al., 2017). Conversely, it is argued, the absence of developmental impairment may be associated with continuing on a pathway to affective psychosis. These differences in illness trajectory, mediated by the neurodevelopmental correlates of city living, could help explain the specificity of the urbanicity association to non-affective psychosis. The converse finding, that associations with neighbourhood ethnic density were similar for both disorders, may reflect a shared aetiological pathway. For example, it is hypothesised that access to social support is an important mediating factor for the effect of ethnic density (Bécares et al., 2018) and this is implicated as a risk factor for both affective and non-affective psychoses (Alloy et al., 2005; Kirkbride et al., 2008). One exception in our study findings is the experience of European (non-Scandinavian) migrants. It is possible that this different pattern reflects a qualitatively different experience of neighbourhood ethnic density for this group, or simply a much greater heterogeneity in their experience, compared to migrants from Africa and the Middle East.

### 4.5 Implications

When we looked at the association between neighbourhood at age 15 and later psychosis, we found that overall neighbourhood variation was similar for both non-affective and affective psychosis implying similar heterogeneity in rates of both disorders. However, when we looked at specific neighbourhood factors our results were more mixed. Neighbourhood urbanicity was largely associated with non-affective psychosis alone, as previous studies have shown, while neighbourhood ethnic density was typically associated with both types of psychosis. In line with previous studies, our findings therefore support the role of both shared and non-shared environmental risk factors for affective and non-affective psychosis phenotypes. In line with previous studies our findings support the role of both shared and non-shared environmental risk factors for affective and non-affective psychosis phenotypes (Laursen et al., 2007; Cardno and Owen, 2014; Reininghaus et al., 2016).

These differences imply different underlying mechanisms behind these environmental effects. Future studies may benefit from comparing effects across further sub-categories of psychotic disorder where possible.

## Role of the funding source

This work was supported by a UK 10.13039/501100000265Medical Research Council fellowship (MR/K021494/1) to P.S.

## Declaration of competing interest

The study authors have nothing to disclose.

## Appendices Supplementary data

Appendix 1 ICD-8 and ICD-10 codes used to determine affective psychosis. Appendix 1

Appendix 2 Sensitivity analysis part 1. Appendix 2

Appendix 3 Appendix 3

Appendix 4 Country of origin categories. Appendix 4

Appendix 5 Frequency of psychotic disorder. Appendix 5
