# Supplementary material for: A comparison of neighbourhood level variation and risk factors for affective versus non-affective psychosis
Source: Schizophr Res. 2023 Jun;256:126–32. doi: 10.1016/j.schres.2022.05.015 (PMC10259518; doi:10.1016/j.schres.2022.05.015)
Supplement: Appendix 1 — ICD-8 and ICD-10 codes used to determine affective psychosis. [file mmc1.docx]

**Appendix 1**

### ICD-8 and ICD-10 codes used to determine affective psychosis

Personal communication from Aksel Bertelsen – dated 25th June 2015:

“F30-33 does not contain affective psychoses, only F30.2, F31.2, F31.5, part F31.6, part F31.8, F32.3 and F33.3 contain affective disorders with psychotic symptoms (hallucinations and delusions).

ICD-8 used the former concept of psychosis with deceptive sense of reality and adaptation to reality and includes 296 as well as 298.0 and 298.1, all of which could be with or without psychotic symptoms.

The above ICD-10 and ICD-8 diagnoses are probably the best possible approximation one can get.”
