# Supplementary material for: A comparison of neighbourhood level variation and risk factors for affective versus non-affective psychosis
Source: Schizophr Res. 2023 Jun;256:126–32. doi: 10.1016/j.schres.2022.05.015 (PMC10259518; doi:10.1016/j.schres.2022.05.015)
Supplement: Appendix 2 — Sensitivity analysis part 1. [file mmc2.docx]

## Appendix

Table 5. Variation in incidence of psychotic disorder across neighbourhoods^a^
– excluding cases where diagnosis changed within five years of the first diagnosis

|  | Median Risk Ratio (95% CI) | |
| --- | --- | --- |
|  | Non-Affective Psychosis | Affective psychosis |
| Model 1^b^ | 1.37 (1.34 – 1.40) | 1.46 (1.40 – 1.52) |
| Model 2^c^ | 1.31 (1.28 – 1.33) | 1.47 (1.41 – 1.54) |

^a^where cohort member was resident at age 15
^b^adjusted for age, gender and calendar period
^c^adjusted for age, gender, calendar period, ethnicity, parental psychiatric history and parental income

Table 6. Incidence rate ratios of psychotic disorder by neighbourhood^a^ urbanicity
– excluding cases where diagnosis changed within five years of the first diagnosis

|  | Incidence rate ratio (95% CI)^b^ | |
| --- | --- | --- |
| Urbanicity (quintile) | Non-Affective Psychosis | Affective psychosis |
| 1 (lowest) | 1 | 1 |
| 2 | 1.07 (1.01 - 1.13) | 0.97 (0.86 - 1.10) |
| 3 | 1.22 (1.15 - 1.29) | 0.99 (0.87 – 1.12) |
| 4 | 1.30 (1.23 - 1.38) | 0.94 (0.82 - 1.07) |
| 5 (highest) | 1.66 (1.57 - 1.75) | 1.13 (1.00 - 1.28) |

^a^where cohort member was resident at age 15
^b^adjusted for age, gender, calendar period, parental psychiatric history and income

Table 7. Incidence rate ratios of psychotic disorder by neighbourhood^a^ ethnic density for each migrant group – excluding cases where diagnosis changed within five years of the first diagnosis

|  | Incidence rate ratio (95% CI)^b^ | |
| --- | --- | --- |
| Ethnic density trend (across quintiles) for each country of origin^c^ | Non-Affective Psychosis | Affective psychosis |
| Africa | 1.24 (1.10 – 1.39) | 1.33 (0.86 – 2.05) |
| Europe | 1.15 (1.08 – 1.21) | 0.99 (0.83 – 1.18) |
| Middle East | 1.14 (1.05 – 1.24) | 1.66 (1.25 – 2.21) |

^a^*where cohort member was resident at age 15*^b^*adjusted for age, gender, calendar period, parental psychiatric history and income and neighbourhood urbanicity*
^c^*measured as trend over decreasing ethnic density quintiles i.e. the average change in psychosis incidence corresponding to a change from one ethnic density quintile to the next lower density quintile*
