# Supplementary material for: A comparison of neighbourhood level variation and risk factors for affective versus non-affective psychosis
Source: Schizophr Res. 2023 Jun;256:126–32. doi: 10.1016/j.schres.2022.05.015 (PMC10259518; doi:10.1016/j.schres.2022.05.015)
Supplement: Appendix 3 [file mmc3.docx]

## Appendix 3

Table 8. Incidence rate ratios of psychotic disorder by neighbourhood^a^ urbanicity. Restricted to individuals with follow-up in the ICD-10 period only.

|  | Incidence rate ratio (95% CI)^b^ | |
| --- | --- | --- |
| Urbanicity (quintile) | Non-Affective Psychosis | Affective psychosis |
| 1 (lowest) | 1.00 (ref) | 1.00 (ref) |
| 2 | 1.05 (0.99 - 1.11) | 0.97 (0.87 - 1.09) |
| 3 | 1.18 (1.11 - 1.26) | 0.98 (0.87 - 1.11) |
| 4 | 1.26 (1.19 - 1.35) | 0.94 (0.83 - 1.07) |
| 5 (highest) | 1.60 (1.50 - 1.69) | 1.15 (1.02 - 1.29) |

^a^where cohort member was resident at age 15
^b^adjusted for age, gender, calendar period, parental psychiatric history and income

Table 9. Incidence rate ratios of psychotic disorder by neighbourhood^a^ ethnic density
for each migrant group. Restricted to individuals with follow-up in the ICD-10 period only.

|  | Incidence rate ratio (95% CI)^b^ | |
| --- | --- | --- |
| Ethnic density trend (across quintiles) for each country of origin^c^ | Non-Affective Psychosis | Affective psychosis |
| Africa | 1.20 (1.07 - 1.34) | 1.47 (1.04 - 2.09) |
| Europe | 1.16 (1.09 - 1.22) | 1.02 (0.88 - 1.20) |
| Middle East | 1.14 (1.05 - 1.25) | 1.57 (1.21 - 2.03) |

^a^*where cohort member was resident at age 15*

^b^*adjusted for age, gender, calendar period, parental psychiatric history and income and neighbourhood urbanicity*
^c^*measured as trend over decreasing ethnic density quintiles i.e. the average change in psychosis incidence corresponding to a change from one ethnic density quintile to the next lower density quintile*
