# Supplementary material for: A comparison of neighbourhood level variation and risk factors for affective versus non-affective psychosis
Source: Schizophr Res. 2023 Jun;256:126–32. doi: 10.1016/j.schres.2022.05.015 (PMC10259518; doi:10.1016/j.schres.2022.05.015)
Supplement: Appendix 4 — Country of origin categories. [file mmc4.docx]

### Appendix 4. Country of origin categories

In the statistical analysis country of origin is organised into three mutually exclusive categories:

1. Africa – any country in the African continent: Algeria, Angola, Asmara, Benin, Botswana, Burkina Faso, Burundi, Cameroon, Cape Verde, Central African Republic, Comoros, Democratic Republic of the Congo, Djibouti, Egypt, Ivory Coast, Ethiopia, Eritrea, Gabon, Gambia, Ghana, Guinea Bissau, Guinea, Kenya, Lesotho, Liberia, Libya, Madagascar, Malawi, Mali, Morocco, Mauritania, Mauritius, Mozambique, Namibia, Niger, Nigeria, Republic of the Congo, Rwanda, Sao Tome and Principe, Senegal, Seychelles, Sierra Leone, Somalia, Sudan, Swaziland, South African Republic, South Sudan, Tanzania, Chad, Togo, Tunisia, Uganda, Zimbabwe, Zambia, Equatorial Guinea.
2. Europe – any country in the European continent excluding Scandinavian countries (Denmark, Norway, and Sweden): Albania, Andorra, Belarus, Belgium, Bosnia and Herzegovina, Bulgaria, Cyprus, Estonia, France, Greece, Ireland, Italy, Yugoslavia/Socialist Federal Republic of Yugoslavia, Kosovo, Croatia, Latvia, Liechtenstein, Lithuania, Luxembourg, Macedonia, Malta, Moldova, Monaco, Montenegro, The Netherlands, Poland, Portugal, Romania, Russia, San Marino, Switzerland, Serbia, Serbia & Montenegro, Slovakia, Slovenia, Soviet Union, Spain, United Kingdom, Czech Republic, Czechoslovakia, Turkey, Germany/Federal Republic of Germany, Ukraine, Hungary, Vatican City State, Austria.
3. Middle East – Afghanistan, Bahrain, United Arab Emirates, Israel, Iraq, Iran, Jordan, Kuwait, Lebanon, Palestinian National Authority, Oman, Qatar, Saudi Arabian, Syria, Republic of Yemen.
