# Supplementary material for: A comparison of neighbourhood level variation and risk factors for affective versus non-affective psychosis
Source: Schizophr Res. 2023 Jun;256:126–32. doi: 10.1016/j.schres.2022.05.015 (PMC10259518; doi:10.1016/j.schres.2022.05.015)
Supplement: Appendix 5 — Frequency of psychotic disorder. [file mmc5.docx]

## Appendix 5

Table 10. Frequency of psychotic disorder

| Country of origin | Non-Affective Psychosis | Affective psychosis | Total (N) |
| --- | --- | --- | --- |
| Denmark | 24,444 | 4,550 | 28,994 |
| Africa | 363 | 34 | 397 |
| Europe | 1,175 | 138 | 1,313 |
| Middle East | 531 | 69 | 600 |
